# Establishment of stable Huh-7 cell lines expressing various hepatitis C virus genotype 3a protein: an in-vitro testing system for novel anti-HCV drugs

**DOI:** 10.1186/1479-0556-9-12

**Published:** 2011-06-28

**Authors:** Sadia Butt, Muhammad Idrees, Irshad-ur Rehman, Liaqat Ali, Abrar Hussain, Muhammad Ali, Naveed Ahmed, Sana Saleem, Madiha Fayyaz

**Affiliations:** 1 Molecular Virology Laboratory, National Centre of Excellence in Molecular Biology, 87-West Canal Bank Road ,Thokar Niaz Baig, Lahore-53700, University of the Punjab, Lahore, Pakistan

## Abstract

**Background:**

Hepatitis C virus (HCV) infection is the leading cause of chronic hepatitis which progresses to hepatocellular carcinoma (HCC) afflicting > 170 million people worldwide. HCV 3a is the most common genotype (about 70% of all genotypes) circulating in Pakistan. Expression of HCV individual gene of 3a would facilitate therapeutic and vaccines strategies against chronic HCV and liver Cirrhosis. The aim of the present study was the establishment of stable Huh-7 cell lines expressing structural and non structural proteins of HCV Genotype 3a Pakistani isolate obtained from chronic HCV patients.

**Methods:**

Blood samples were obtained from chronic HCV-3a positive patients. HCV individual genes were amplified using PCR with gene specific primers having restriction sites. These gene amplicons were cloned in mammalian expression vector PcDNA3.1+. Huh-7 cell lines were transfected with these constructed plasmids having structural or non-structural HCV genes in confluent cells with lipofectamine. Positive clones were selected with G418 and then confirmed by genome PCR. Subsequently, transcription and expression of the integrated genes were demonstrated by RT-PCR, sequencing and Western blot analysis.

**Results:**

We successfully cloned and express five HCV-3a genes in PcDNA3.1+ mammalian expression vector. Results of western blot and sequencing PCR confirmed the stable expression of these five genes.

**Conclusion:**

The stable cell-lines expressing HCV-3a individual genes would be a useful tool to investigate the role of various HCV proteins on HCV disease outcome and testing of new therapeutic strategies against HCV.

## Background

Hepatitis C virus (HCV) is an enveloped plus-strand RNA virus of family *Flaviviridae *[1,2]. HCV is a major leading cause of chronic liver disease [3]. An estimated 170-200 million persons worldwide are infected with HCV [4-6]. Studies on virus replication and pathogenesis having difficulties due to the unavailability of consistent and efficient cell culture systems, even though increasing knowledge of genome structure and individual viral proteins [7]. The HCV genome is approximately 9.6 kb in length and consists of a single open reading frame (ORF) encoding a polyprotein of about 3,000 amino acids and un-translated regions (UTRs) located at the 5'and 3' terminus of the genome [8,9].

At the 5' end HCV genome there are structural genes; the nucleocapsid region core (C), and the envelope regions (E1 and E2). The 5' UTR and C are conserved regions, while the envelope domain E2/NS1 encloses the hyper variable region [10,11]. After the C gene towards the 3' end, are six non-structural regions (NS2, NS3, NS4A, NS4B, NS5a & NS5B) [7,12]. Viral proteins included in various immunoassays and in the recombinant immunoblot assay are presented below their corresponding genes [13]. HCV does not integrate into the host genome as it does not replicate via a DNA intermediate. Even if the in-vitro HCV replication remains a challenge, the chimpanzee is the only important experimental animal model [13]. At the 5'UTR, an internal ribosome entry site (IRES) is located where in a cap-independent manner, viral proteins are expressed. There are 10 viral proteins: core; envelope protein 1 (E1) and E2 are structural proteins that constitute the virion; a small protein that is essential for protein assembly [10,11,14] and six non structural proteins (NS2, NS3, NS4A/B and NS5A/B).

The core protein of HCV forms the nucleocapsid of the virus. It binds with RNA and also interacts with numerous cellular proteins. Various host cell functions such as gene transcription, lipid metabolism, apoptosis and certain signaling pathways are also reported to have interaction with core protein [15]. The associations of core protein with the induction of steatosis and HCC have also been reported [16]. HCV core Ag proved to be useful for performing HCV RNA measurement among dialysis patients in routine laboratories without the need for special equipment or training [17].

E1 protein is associated with the membrane fraction [18]. A direct role for the C-terminal domain in E1 membrane association was identified in the soluble phase by the truncated mutant E1t [19]. The HCV E1 protein having good specificity and could be used in the diagnosis of HCV infection [20] can become useful tools in anti-HCV vaccine research [21]. The NS2 protein is a 23-kDa hydrophobic transmembrane, anchored to the endoplasmic reticulum (ER) [22,23] its function is reliant on the microsomal membranes occurrence, but the function of the NS2 protein in cells is still very poorly understood [24,25]. It has been found that the HCV NS2 protein inhibits cell proliferation and induces cell cycle arrest in the S-phase in mammalian cells through down-regulation of cyclin A expression [24]. Nonstructural protein 4A (NS4A) is a multifunctional protein with 54 amino acid residues. It acts as a cofactor of NS3 serine protease and plays an essential role in the NS4A-dependent cleavage at the NS3-NS4A and NS4B-NS5A junctions [26,27]. Both NS4A and NS4B proteins were previously demonstrated to suppress translation in culture cells [28,29]. HCV NS4B is a highly hydrophobic, localized to the endoplasmic reticulum (ER) and induces a pattern of cytoplasmic foci positive for markers of the ER through four transmembrane segments [30]. NS4B is also a helper factor for the HCV RNA dependent RNA polymerase suggested by the mutagenesis studies of the nucleotide binding motif of NS4B [31]. The involvement of HCV NS4B in IFN-alpha resistance was also reported by some groups [32,33]. However no such study is available on the construction of these expressions vectors from Pakistan where the rate of HCV is 8-10% in general population and novel and chief drugs are required to treat so huge number of cases.

Therefore, in this study, we have constructed five expression vectors encoding structural (core and envelope1) and nonstructural (NS2, NS4A, NS4B) genes from local HCV isolates and checked their stable expression in Huh-7 cell line. These expression vectors have the potential to be use for testing of new developed drugs in cell culture system.

## Methods

### Sample collection

Chronic HCV infected with Genotype 3a positive samples were obtained from Division of Molecular Virology and Molecular Diagnostics, National Centre of Excellence in Molecular Biology (CEMB), Lahore, Pakistan. HCV genotyping was carried out on positive HCV PCR samples using type specific HCV genotyping methods as described previously [34,35].

### Construction of plasmid (HCV genes in mammalian expression vector PcDNA3.1+)

From the HCV positive serum with 3a genotype, RNA was extracted using Gentra RNA isolation kit (Gentra System Pennsylvania, USA) and individual gene is reverse transcribed using M-MLV (Invitrogen Life technologies, CA). HCV reference sequence of NZL1 # D17763 was used for primer designing on Primer 3 software, restriction sites and kozak sequences were added after restriction analysis on web cutter and neb-cutter primers sequences given in table 1. Each gene is amplified individually and completely. Amplified genes with restriction sites were then cloned in mammalian expression vector PcDNA3.1+ (Invitrogen Life technologies, CA). Each gene constructed plasmid were confirmed through PCR, restriction digestion and sequenced. Individual gene sequence submitted to genbank accession numbers given in table 2.

**Table 1:** indicating HCV Gene and polyprotein sequences submitted in Genbank and their Accession Numbers

*No.*	*HCV Gene and polyprotein*	*Sequences*	Accession Numbers
1.	Core	Hepatitis C virus isolate PKIS-1 polyprotein gene, partial cds.	FJ851546.2
		Hepatitis C virus isolate PKIS-2 core polyprotein gene, partial cds.	HQ323687
		Hepatitis C virus isolate PK-1 complete genome.	GU294484.1
2.	Envelope 1	Hepatitis C virus genotype 3a isolate PKIS-2 e1 complete polyprotein gene.	HQ433527
		Hepatitis C virus isolate PK-1 complete genome	GU294484.1
3.	Non-Structural 2	HCV genotype 3a Non-Structural2 NS2 region of Pakistani isolate.	FJ865505
		Hepatitis C virus clone 3a nonstructural protein 2 Pakistani isolate PKIS-2 polyprotein.	HQ822055
		Hepatitis C virus isolate PK-1 complete genome	GU294484.1
4.	Non-structural 4a	Hepatitis C virus isolate PKIS-1 non structural 4a polyprotein gene, partial cds.	HQ822054
		Hepatitis C virus isolate PK-1 complete genome.	GU294484.1

**5.**	Non-structural 4b	Hepatitis C virus isolate PK1 non-structural protein NS4b gene, partial cds.	GQ325251
		Hepatitis C virus isolate PKIS-2 non-structural protein NS4b gene, partial cds.	HQ323685
		Hepatitis C virus genotype 3a PKIS-3 non-structural protein NS4b.	HQ433528
		Hepatitis C virus genotype 3a isolate PKIS-4 non-structural protein 4b.	HQ616144
		Hepatitis C virus isolate PK-1 complete genome	GU294484.1

**Table 2:** List of primers of each individual gene of HCV genotype 3a, Restriction sites worked successfully, Nucleotide position in full length sequence reference sequence of NZL1 was used and number of nucleotides in each amplified region

No.	Genes	Primer seq. 5'-3'	Restriction site	No. of Nucleotides (amplified region)
**1.**	CORE-IS	ATGAGCACACTTCCTAAACCTCA	*Hind III*	
	
**2.**	CORE-IAS	ACTGGCTGCTGGATGAATTAAGC	*EcoR1*	**573**

**3.**	E1-IS	CTAGAGTGGCGGAATACGTCTG	*Hind III*	
	
**4.**	E1-IAS	GGCGACCCCTGAGAACATAACC	*EcoR1*	**576**

**5.**	NS2-IS	CTTTGGTCCCTAGCATTGC	*Hind III*	
	
**6.**	NS2-IAS	CCTCACGGCCTAATCGTGC	*EcoR1*	**642**

**7.**	NS4A-IS	AGCACCTGGGTGTTGCTC	*Hind III*	
	
**8.**	NS4A-IAS	GCACTCCTCCATCTCATCGT	*EcoR1*	**168**

**9.**	NS4B-IS	TCACAAGCTGCCCCATATATCG	*Hind III*	
	
**10.**	NS4B-IAS	GCTACAAGGGCTTGGGTAGTC	*Xba1*	**783**

### Cell culture and transfection

Huh-7 cell lines were used maintained in Dulbecco's modified eagle medium (DMEM) supplemented with 100 μg/ml penicillin; streptomycin and 10% fetal bovine serum (Sigma Aldrich, USA) at 37°C with 5% CO2. cells were seeded in 24-well (1 × 10^5^/well) or 6- well (5 × 10^5^/well) plates and cultured until they became 70-80% confluent. Constructed plasmids about 3-4 ug of structural (core and E1) and non-structural (NS2, NS4A, NS4B) HCV genes were transfected in confluent cells with lipofectamine (Invitrogen Life technologies, CA) after 6-8 hrs. of transfection media (with lipofectamine and plasmid) was changed.

### Isolation of RNA

RNA was isolated using Gentra Kit and reverse transcribed to cDNA with reverse primer and specific genes were amplified with gene-specific primers for mRNA confirmation.

### Proteins extraction and Immuno-blot (Western blotting)

Cells were lysed and protein was extracted after 72 hrs. after transfection and for single stable clones after 3 weeks in PLB (150 mM6/29/2011 NaCl, 1M Tris-Cl pH 7.4, 5 mM EDTA, 1% Triton X-100) proteinase inhibitor and 1 mM PMSF, kept on ice for 15 min. 80-100 μg of total protein were loaded in each well on 10-12.5% SDS-PAGE gels and electrophoretically blotted onto a Hybond-C extra nitrocellulose membrane semi-dry blotting apparatus (Bio-Rad). The membrane was blocked for 1 hour with a 5% milk solution in Phosphate Buffered Saline-0.05% Tween (PBS-T), washed three times with 50 ml of PBS-T. A mixture of primary antibodies for structural genes like core (sc-57800), E1 (sc-65459) and non structural gene like NS4A (sc-52415), NS4B (sc-65457) was added, each at a concentration of 1:500-1:800 in 5 ml of PBS-T. After incubating at room temperature for 1 hour, the membrane was washed 3 times with PBS-T. A secondary antibody, rabbit anti-mouse IgG, conjugated to alkaline phosphatase (Sigma), was added at a dilution of 1/1000 in PBS-T, incubated at room temperature for one hour. The membrane was washed for three times with PBS-T. Substrate tablet (NBT/BCIP) was dissolved in 1XPBS and blot was incubated for 15-30 min.

### Generation of stable cell lines of structural and non structural proteins

After 72 hrs of transfection, cells were given selection with G418 initially with 400 ug/ml for selecting stable clones than after 14 days were given 200 ug/ml. The medium was changed after every 72 hours day. Colonies of G418 resistant cells were selected and grown further and confirmed with PCR, western blotting and sequencing.

## Results

**Figure 1:**
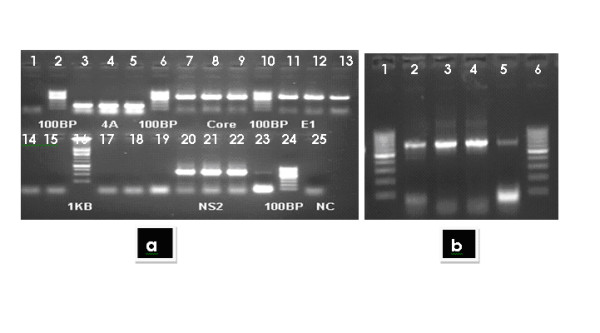
**a) Showing the amplified genes of Core (573), Envelope 1(576), Non-structural 2 (NS2, 642 bp) and Non Structural 4a (NS4A, 168 bp), b) lanes 2-5 (left to right) showing the complete amplified region of 783 bp of Non Structural 4b gene**. *From the HCV positive serum with 3a genotype, RNA was extracted and individual gene was reverse transcribed using M-MLV. HCV reference sequence of NZL1 # D17763 was used for primer designing on Primer 3 software, restriction sites and kozak sequences were added after restriction analysis on web cutter and neb-cutter primers sequences. Each entire gene was amplified individually. Amplified genes with restriction sites were then cloned in mammalian expression vector PcDNA3.1+*.

**Figure 2:**
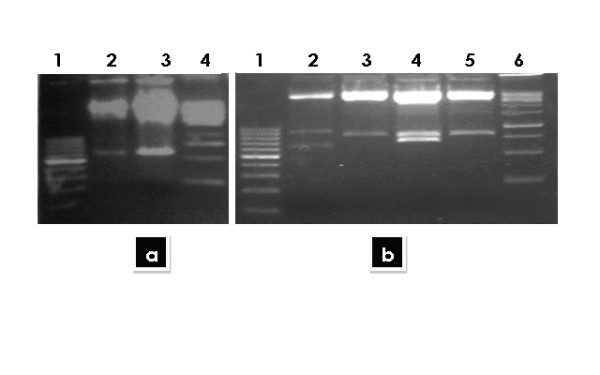
**(a) Digestion of Structural genes (Core and E1):**. *Lane 1 showing 100-bp Marker; lane 2 and 3 showing digestion of Core and E1 genes; lane 4, showing 1 kb ladder*. (b) Digestion of Non-structural genes (NS2, NS4b): *Lane 1 showing 100-bp Marker; lane 2 and 4 digestion of NS2; lane 3 and 5 showing digestion of Ns4b; lane6: showing 1 kb ladder*.

Figure 1 (a & b) showing amplified structural (core and envelope1) and nonstructural (NS2, NS4A, NS4B) genes of the exact sizes. These bands were confirmed by sequencing and only the sequence confirmed genes were further used in next experiment leading to the development of expression vectors. The genes were then cloned in mammalian expression vector pcDNA 3.1+. The successful clones of these genes in PcDNA3.1+ vector were confirmed using restriction digestion analysis. The results of restriction digestions are shown in figure 2. This vector has a CMV promoter which represents an effective mean to transduce eukaryotic cells for transient and stable expression studies. The cloned genes were sequenced in both direction and the consensus sequence was matched to HCV genotype 3a sequence when blast was done with other HCV sequences in GenBank data base.

The expression vector was then linearized and transfected into Huh7 cells by lipofectamine. Twenty-four hours post transfection, selection was applied to the transfected cells by growing them in the presence of 1 mg of G418/ml. About 80% of cells did not develop resistance to the selecting agent, but in the long run it was possible to identify G418-resistant cell clones, which were picked after four weeks of culture and grown as individual cell lines. Once the clones had been isolated and individually grown as cell lines, the concentration of neomycin was decreased to 500 μg/ml. The individual cell lines showed some variability in growth rate.

**Figure 3:**
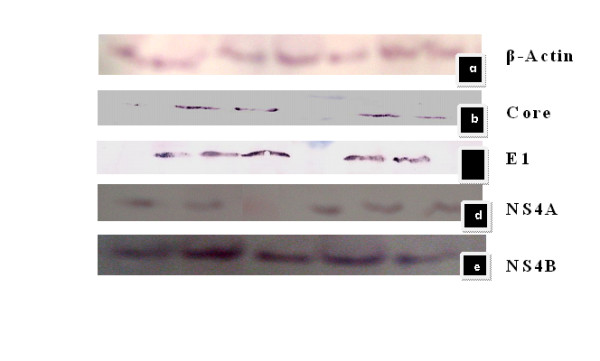
**(Top to Bottom) a: blot result of positive control B-Actin; b, c, d, and e are blot results of Core, E1, NS4A, NS4B respectively developed by AP conjugated Anti mouse with NBT/BCIP substrate (sigma)**. *Cells were lysed and protein was extracted after 72 hrs after transfection for single stable clone after 3 weeks About 80-100 μg of total protein were loaded into each well on 12.5% SDS-PAGE and electrophoretically blotted onto a Hybond-C extra nitrocellulose membrane semi-dry blotting apparatus. The membrane was blocked for 1 hour with a 5% milk solution in Phosphate Buffered Saline-0.05% Tween (PBS-T), washed three times with 50 ml of PBS-T. A mixture of primary antibodies for structural genes core (sc-57800), E1 (sc-65459) and non structural gene NS4A (sc-52415), NS4B (sc-65457) was added at a concentration of 1:500-1:800 in 5 ml of PBS-T. After incubating at room temperature for 1 hour, the membrane was washed 3 times with PBS-T. A secondary antibody, rabbit anti-mouse IgG, conjugated to alkaline phosphatase was added at a dilution of 1/1000 in PBS-T, incubated at room temperature for one hour. The membrane was washed for three times with PBS-T. Substrate tablet (NBT/BCIP) was dissolved in 1XPBS and blot was incubated for 15-30 min*.

To check expression of various HCV individual proteins produced from corresponding replicon clones, we performed Western blot analyses with protein extracts of transfected Huh-7 cells. Figure 3 showing the western blot results of structural and non structural proteins. The Western blot analysis identified specific bands of the expected electrophoretic mobility. B-Actin was used as a positive control. Antibodies of NS2 are not available so it was proceed the same way that was confirmed by sequence analysis and RT-PCR confirmed it. The expression of these individual genes were confirmed by RT PCR and sequencing. All the sequences were submitted to Genbank data base. Table 1 indicating HCV Gene, polyprotein sequences submitted to Genbank data base and their assigned Accession Numbers. Table 2 shows the list of primers of each individual gene of HCV genotype 3a, restriction sites worked successfully, Nucleotide position in full length reference sequence of NZL1 was used and number of nucleotides in each amplified region.

## Discussion

Despite vigorous host immune response, 20% of those infected with chronic HCV will eventually lead to HCC [36]. The socio-economic burden of HCV infection globally is striking with an urgent necessity to have better information of viral pathogenesis in order to develop new anti-HCV strategies.

To test novel drugs for its inhibitory action, an efficient culture system is required for the amplification of virus. To date an efficient and reliable culture system is not available to amplify HCV [2] and this limitation prevents the elaboration of reliable infection assays. Several models based on the self-replication of engineered mini-genomes in cell cultures, has been established for HCV replication in other regions of the world [7,37]. The HCV stable cell lines may be very useful in the study of HCV genomic replication in that part of the world where other HCV genotypes exist. As HCV genotype 3a is the predominant genotype circulating in Pakistan [34,38], therefore, new approaches based on this local HCV genotype 3a are needed on urgent basis to study HCV assembly and infection to design HCV cell entry inhibitors and further to study the humoral immune response against HCV. Therefore, we have developed cell-culture based systems stably expressing two structural and three non-structural HCV individual genes described in the current study.

To the best of our knowledge no cell culture based system has yet been developed to propagate the replication expression of HCV 3a genes of Pakistani chronic isolates in cultured mammalian cells. Because the existing replicon was generated using genotype 1b HCV RNA, the present replicon system may not be used to detect responses that are genotype and subtype-dependent. Therefore this study was initiated to establish stable cell lines expressing proteins of Pakistani HCV genotype 3a isolates. The establishment of HCV genotype 3a cell lines stably expressing structural and non structural proteins is an instrumental in the further study of HCV replication and viral-host interaction of genotype 3a. Viral and cellular factors required for HCV replication will be defined by cutting edge gene and micro-array, proteomics, protein-protein interactions methodologies. Further investigation on these stable cell lines must have direct impact on HCV disease outcome and new therapeutic strategies will be designed.

## Conclusion

In summary, we were able to develop vectors stably expressing HCV individual proteins such as core, envelope1 (Structural), NS2, NS4A and NS4B (Non-structural). The stable cell line expressing individual HCV gene would be useful in identifying the role of most important genes in HCC and fibrosis development and studying the mechanisms of each gene in HCV replication. Obviously, novel therapeutic strategies are required on urgent basis as the health costs for HCV-infected people are predicted to spiral dramatically in the next decade. Further investigation on these stable cell lines must have direct impact on HCV disease outcome and new therapeutic strategies will be designed. This system with genes from HCV-3a strain can be used for comparison studies with other strain-derived systems developed in other areas for the analysis of the effects of anti-HCV drugs.

## Competing interests

The authors declare that they have no competing interests.

## Authors' contributions

SB and IR reviewed the literature, conducted all the experiments and wrote the manuscript. MI guided conducting the whole experiment and edited the manuscript. LA, MA, AH, BR, SS, NA, helped SB & IR in literature review. All the authors read and approved the final manuscript.
